# Characterization of carotenoids in *Lycium barbarum* fruit by using UPC^2^-PDA-Q-TOF-MS^E^ couple with deep eutectic solvents extraction and evaluation of their 5α-reductase inhibitory activity

**DOI:** 10.3389/fchem.2022.1052000

**Published:** 2022-11-08

**Authors:** Zhonglian Yu, Mengqin Xia, Xueping Li, Rui Wang, Wenjing Liu, Ruirong Zheng, Zhengtao Wang, Li Yang, Yanhong Shi

**Affiliations:** ^1^ School of Pharmacy, Shanghai University of Traditional Chinese Medicine, Shanghai, China; ^2^ Institute of TCM International Standardization, Shanghai University of Traditional Chinese Medicine, Shanghai, China; ^3^ The MOE Key Laboratory of Standardization of Chinese Medicines, Institute of Chinese Materia Medica, Shanghai University of Traditional Chinese Medicine, Shanghai, China; ^4^ Institute of Interdisciplinary Integrative Medicine Research, Shanghai University of Traditional Chinese Medicine, Shanghai, China

**Keywords:** *Lycium barbarum* fruit, carotenoids, deep eutectic solvents, UPC^2^-PDA-Q-TOF-MS^E^, 5α-reductase inhibitory activity, zeaxanthin dipalmitate

## Abstract

Carotenoids from *Lyciu* *m barbarum* fruits have possessed pharmacological efficacy against eye diseases, cardiovascular disorders, cancer, and benign prostatic hyperplasia. However, the efficient extraction, rapid characterization and activities evaluation of *Lycium* carotenoids remains a challenge. To concentrate and characterize *Lycium* carotenoids, we have developed ultrasound-assisted extraction methods with different deep eutectic solvents (DESs) and analyzed carotenoids by ultra-performance convergence chromatography coupled with photo diode array detector and quadrupole time-of-flight mass spectrometry (UPC^2^-PDA-Q-TOF-MS^E^). DESs containing choline chloride and malonic acid presented better extraction efficiency and were more environmentally friendly than other extraction methods. Carotenoids were more quickly profiled (in 11 min) by UPC^2^ compared to by UPLC (in 35 min), with seventeen main peaks were characterized in the MS fragmentation patterns. The *in vitro* 5α-reductase inhibitory activity of DESs extracts, fractions and components were subsequently assessed, and the predominant component zeaxanthin dipalmitate (ZD) exhibited potent inhibitory activity. Our study provides a chemical and pharmacological basis for the further development of potential new drugs based on *Lycium* carotenoids.

## Introduction

*Lycium barbarum* fruit is derived from the dry ripe fruit of *L. barbarum* L. (i.e., goji berries) in the family Solanaceae, which possesses potent medicinal effects due to an abundance of bioactive chemical constituents, such as flavonoids, polysaccharides, alkaloids, and carotenoids (Wang et al., 2018). Carotenoids, which are responsible for the red color of the fruits, are relatively rich in content and represent one of the most important nutritional components of *L. barbarum* fruit, playing a role in the prevention of cancer, cardiovascular diseases, certain eye and liver diseases and in the treatment of prostate-related diseases as a 5α-reductase inhibitor ((Anderson, 2005; Peng et al., 2005; Fernández-García et al., 2012; Saini et al., 2015; Wang et al., 2015; Russo et al., 2016). However, studies on efficient extraction, rapid characterization and activities evaluation of *Lycium* carotenoids are limited.

Carotenoids containing eight isoprenoid units with a conjugated double-bond system are susceptible to oxidation and *cis-trans*-isomerization, especially when exposed to light, heat and acid, which makes it particularly challenging to efficiently extract and characterize these ingredients in natural foods and traditional Chinese medicines (TCMs) (Provesi et al., 2011; Amorim-Carrilho et al., 2014). At present, although several extraction methods including ultrasound-assisted extraction, pressurized liquid extraction, enzyme-assisted extraction and supercritical fluid extraction (SFE) have been reported to obtain carotenoids from foods and TCMs, there are several limitations since they require long time, expensive instrumentation and enzymes as well as large amounts toxic solvents but provide low yields (Adadi et al., 2018; Saini and Keum, 2018). Additionally, analytical methods, such as high-performance liquid chromatography (HPLC) coupled with a diode-array-detector or mass spectrometry (MS), produce a poor separation despite of the hazardous mobile phase reagents and extended run durations (Rivera and Canela-Garayoa, 2012; Amorim-Carrilho et al., 2014). More importantly, the absence of high purity reference substances makes it difficult to determine *Lycium* carotenoids content rapidly and accurately for its quality evaluation. Therefore, it’s urgent to develop a more efficient and greener method for extraction and analysis about *Lycium* carotenoids.

The concept of “green chemistry” has become a tool for promoting sustainable development in laboratories and industries. The applications of non-toxic and environmentally green solvents like ionic liquids have been promising and garner more attention (Saini and Keum, 2018). Deep eutectic solvents (DESs), a kind of emerging green solvent, are commonly produced by mixing two or more naturally available, inexpensive and biodegradable constituents, which interact primarily through hydrogen bonding and Van der Waals interactions, making the melting point of deep-eutectic mixtures considerably lower than before mixing (Hadi et al., 2015; Fernández et al., 2018; Fernández et al., 2018). Owing to their several advantages such as low toxicity, non-volatile, non-flammable, biodegradable and excellent thermal stability as well as wide liquid ranges, DESs have been extensively applied in the extraction of active ingredients such as flavonoids, anthocyanins and phenolic compounds from TCMs and foods (Fernández et al., 2018; Ali et al., 2019; Alañón et al., 2020; Cañadas et al., 2020). However, the DESs-based extraction of *Lycium* carotenoids has not been reported.

Compared with ultra-performance liquid chromatography (UPLC), ultra-performance convergence chromatography (UPC^2^), an improved supercritical fluid chromatography with higher pressure, smaller solid-phase particles and less organic solvent usage, has been widely used for chemical profiling in foods and TCMs by virtue of a powerful separation and analysis ability, especially for low polarity compounds, such as carotenoids (Bonaccorsi et al., 2016; Pfeifer et al., 2016; Gao et al., 2017; Rathi et al., 2017). Furthermore, quadrupole time-of-flight mass spectrometry (Q-TOF-MS) is an excellent tool to identify complex chemical components in botanical, foods and pharmaceutical samples because of its ability to provide accurate mass-to-charge ratio and fragment information (Zhou et al., 2009). Therefore, UPC^2^-Q-TOF-MS^E^ may be an effective combination to characterize *Lycium* carotenoids.

Benign prostatic hyperplasia (BPH) is a common urinary disease in men and its prevalence is positively correlated with age, which has become an important medical problem with an increasing affected patients. Although the molecular mechanism of BPH development has not been clearly elucidated, the general consensus is that increased steroid 5α-reductase activity is a risk factor for abnormal prostate growth and BPH development (Sun et al., 2011). 5α-Reductase is membrane-bound enzyme in the oxidoreductase family that regulates steroid metabolism (particularly type 1 and type 2 5α-reductase) and irreversibly convert testosterone to dihydrotestosterone (DHT), which has a higher affinity for androgen receptors (Buncharoen et al., 2016; Karnsomwan et al., 2017). When 5α-reductase is overexpressed, excessive DHT will dysregulate the hormone levels in the body, resulting in BPH, male pattern baldness, and acne (Metcalf et al., 1989). Hence, 5α-reductase inhibitors have become first-line treatment for these diseases, and the two most commonly used inhibitors are dutasteride and finasteride (Alisky et al., 2010; Bauman et al., 2014). Unfortunately, these drugs often cause adverse effects that are difficult to alleviate through structural modification of the drugs, including erectile dysfunction, ejaculation issues and decreased libido (Andriole and Kirby, 2003; Traish et al., 2015). Thus, there is a clinical need to look for potential high efficiency and low toxicity 5α-reductase inhibitors in food and TCMs.

In this study, we used UPC^2^ coupled with photo-diode-array detector and quadrupole time-of-flight mass spectrometry (PDA-Q-TOF-MS^E^) to evaluate the distinction between DESs and traditional extraction methods as well as characterize the carotenoids and marker distribution in *L. barbarum* fruit efficiently. Furthermore, the 5α-reductase inhibitory activities of DESs extracts, fractions, and components of *L. barbarum* fruit were evaluated and compared. The results indicate that the developed extraction and analysis methods could be used in the quality and safety assurance of *L. barbarum* fruit, and our study provides a chemical and pharmacological basis for the further development of new drugs based on *Lycium* carotenoids for the prevention and treatment of BPH.

## Materials and methods

### Materials and reagents

*L. barbarum* fruit were purchased from Ningxia Golden Sun Pharmaceutical Co., Ltd. (Ningxia, China), and identified as the fruit of *Lycium barbarum* L. by Prof. Lihong Wu and Dr. Yanhong Shi from Shanghai University of Traditional Chinese Medicine. All-*trans*-*β*-carotene (CAS 7235-40-7; purity ≥98%) and all-*trans*-zeaxanthin (CAS 144-68-3; purity ≥98%) were procured from Chongqing XINHUYU Bio-Technology Co., Ltd. (Chongqing, China). Zeaxanthin dipalmitate (CAS 144-67-2; purity ≥95%) was supplied by YUANYE Bio-Technology. Co., Ltd. (Shanghai, China). The reagents for preparing DESs and organic solvents used for sample extraction were purchased from Sinopharm Group Chemical Reagents Co., Ltd. (Shanghai, China). Reagents for chromatographic analysis were purchased from Thermo Fisher Scientific (MA, United States). Ultrapure water used in the analyses was freshly prepared using a Millipore water purification system (MA, United States). High-purity CO_2_ (99.999%) was purchased from Shanghai LV MIN Gases Co., Ltd. (Shanghai, China). Ammonium formate and ammonium acetate (LC-MS grade) were procured from Sigma-Aldrich (CA, United States).

HaCaT cells line (human keratinocytes) was obtained from the Institute of Biochemistry and Cell Biology (Shanghai, China). Dulbecco’s modified Eagle medium (DMEM; 11995-065), penicillin-streptomycin (PS; 15140-122), 0.25% Trypsin-EDTA (25200-072), fetal bovine serum (FBS; 10099-141), and Dulbecco’s phosphate-buffered saline (DPBS; 14190-144) were procured from Gibco (NY, United States). Nicotinamide adenine dinucleotide phosphate (NADPH) was obtained from Roche Diagnostics GmbH (Mannheim, Germany). Testosterone-d3 and dihydrotestosterone (DHT, purity ≥97.5%) were purchased from Sigma. Testosterone (purity ≥99.5%) was obtained from Dr. Ehrenstorfer GmbH (Augsburg, Germany) and dutasteride (CAS 164656-23-9; purity ≥99%) was from YUANYE Bio-Technology Co., Ltd. Cell Counting Kit 8 (CCK-8, MA0218) was purchased from MEILUN Bio Co., Ltd. (Shanghai, China).

### DESs preparation

DESs contained two or three components, including hydrogen bond donors and acceptors at certain stoichiometric ratios (Table 1), so we prepared 13 DESs, most of which were prepared by heating and stirring until a clear homogeneous liquid was formed. However, DES-10 and DES-11 were formed by freeze-drying (Fernández et al., 2018). These DESs were then used in the extraction of carotenoids from *L. barbarum* fruit without further purification.

**TABLE 1:** The composition of the studied DESs and their abbreviation in this study.

Abbreviations	DESs composition			Molar ratio
	Component 1	Component 2	Component 3	
DES-1	Choline chloride	lactic acid	-	1:1
DES-2	Lactic acid	glucose	water	5:1:1
DES-3	Lactic acid	glucose	water	6:1:6
DES-4	Proline	malic acid	-	1:1
DES-5	Choline chloride	malic acid	-	1:1
DES-6	Proline	glucose	-	5:1
DES-7	Choline chloride	citric acid	-	1:1
DES-8	Choline chloride	lactic acid	-	1:5
DES-9	Choline chloride	malonic acid	-	1:1
DES-10	Citric acid	maltose	-	4:1
DES-11	Proline	glycerol	-	2:5
DES-12	Choline chloride	glucose	-	1:1
DES-13	Choline chloride	glycerol	-	1:2

### Sample preparation

DESs-based extraction: *Lycium* carotenoids were extracted with DESs following a previously published method (Hadi et al., 2015). One gram of crushed *L. barbarum* fruit sample mixed with 2.0 g DES was extracted using 30 ml methanol and 10 ml hexane. After the mixture was subjected to ultrasonication (250 W, 40 KHz) at 20°C for 4 h, the filtrates were mixed with a 10 ml water–hexane mixture (6:4, *v*/*v*) to perform liquid–liquid extraction and repeated three times. Finally, the hexane layer was collected and vacuum dried to obtain concentrate, which was then dissolved in 1.0 ml ethyl acetate and filtered through a 0.22 μm membrane filter for analysis. Importantly, the entire procedure was performed in the dark.

Published extraction methods: reported representative methods were also simultaneously performed to be compared with DESs-based extractions, including traditional extraction through the mixture of organic solvents (hexane-ethanol-acetone-toluene, 10:6:6:7, *v*/*v*/*v*/*v*) and supercritical fluid extraction with supercritical CO_2_ and ethanol as a co-solvent (Inbaraj et al., 2008; Zaghdoudi et al., 2016). These methods were performed as described in the relevant literatures.

### UPLC and UPC^2^ system conditions

UPLC was performed using a Shimadzu LC-30A system (Kyoto, Japan), which was equipped with a system controller, pumps, a diode array detector, an autosampler, and a column oven. An Agilent ZOEBAX RRHD Stable Bond 80Å C_18_ column (150 mm × 2.1 mm, 1.8 μm) was used for carotenoids separation at 40 °C. The mobile phase was composed of ethyl acetate (A) and CH_3_CN-H_2_O (90:10, *v*/*v*; B) with a gradient elution program as follows: 100%–75% B from 0 to 5 min; 75%–55% B from 5 to 10 min; 55%–40% B from 10 to 11 min; 40%–30% B from 11 to 21 min and 30%–30% B from 21 to 35 min. The flow rate was 0.3 ml/min, the injection volume was 3.0 μL and the detection wavelength was set at 450 nm.

UPC^2^ was performed on a Waters ACQUITY UPC^2^ system (Milford, MA, United States) equipped with a binary pump, an auto-sampler, a column oven, a back-pressure regulator and a photodiode array detector. The sample was analyzed on an UPC^2^ HSS C_18_ column (150 mm × 3.0 mm, 1.8 μm) at 35°C. The mobile phases consisted of CO_2_ (A) and methanol (B). The elution started at 20% B, increased linearly to 50% B at 8 min and kept for 3 min, then it went back to the initial concentration and equilibrium for the next injection. The flow rate was 0.8 ml/min. The detection wavelength and reference wavelength were set at 450 nm and 600–700 nm, respectively. The back pressure was set at 1500 psi, and the injection volume was 3.0 μL.

### Q-TOF-MS^E^ conditions

The samples were analyzed using a Waters Synapt-G2 Q-TOF-MS^E^ system combined with a UPC^2^ system. A Waters 1525 single pump was selected as the compensated pump for 5 mM ammonium formate dissolved in methanol at a flow rate of 0.2 ml/min. Data was acquired in positive mode with an electrospray ionization (ESI) source. The data acquisition range was *m*/*z* 100–1200 Da for the MS^E^ mode. The Lockspray™ interface was used for leucine encephalin ([M + H]^+^ *m/z* 556.2766) solution infused at 5.0 μL/min as the lock mass. The capillary voltage was 3.0 kV, sample cone was 20.0 V, and extraction cone was 4.0 V. The source temperature was 120°C and desolvation temperature was 400°C. The cone gas flow rate was 50 L/h and desolvation gas flow rate was 800 L/h. The data was collected in the MS^E^ mode and the collision energy was 10–50 eV. The scan time for each function was set at 0.2 s. Data were collected and analyzed using Waters Masslynx v4.1 software.

### Semi-quantification of *Lycium* carotenoids

For the semi-quantification of *Lycium* carotenoids, the content of zeaxanthin, *β*-carotene and ZD was determined using external standards method. The absolute peak areas of other carotenoids were divided by the peak area of zeaxanthin standard to calculate their relative contents. The final content of each carotenoid is reported as μg per 1.0 g of *L. barbarum* fruit and values are presented as mean ± standard deviation of triplicate experiments.

### Preparation of fractions from the total *Lycium* carotenoids

Semi-preparative liquid chromatography (SPLC) of carotenoids preparation was performed on a LC-3000 system. A YMC-Pack Pro C_18_ column (250 mm × 10.0 mm, I.D.; S-5 μm, 12 nm; AS 12S05-2510WT; Ser. No. 104xA60076, United States) was used. The mobile phases were ethyl acetate (A) and mixed solvent of CH_3_CN - H_2_O (90:10*, v/v*; B). The gradient elution program was as follows: 60% A from 0 to 5 min; 70% A from 5.01 to 25 min; 75% A from 25.01 to 30 min; and 70% A from 30.01 to 40 min. The flow rate was 1.0 ml/min. The detection wavelength was set at 450 nm. The fractions were collected on the basis of main marker distribution as follows: 0–8 min (Fr-1), 8–16 min (Fr-2), 16–24 min (Fr-3), 24–32 min (Fr-4), and 32–36 min (Fr-5).

### HaCaT-based bioassays

Based on our previously developed method (Deng et al., 2020), HaCaT cells were cultured in DMEM containing 10% FBS and 1% PBS (*v/v*) at 37°C in a modified atmosphere containing 5% CO_2_. Cells were seeded in 96-well plates at 1 × 10^4^ cells/well and adhered for 24 h followed by culturing in different treatment concentrations for 24 h. Next, CCK-8 assays were performed to measure cytotoxicity. The medium was replaced with 100 μL CCK-8 in 900 μL DMEM and the absorbance of the mixture was measured at 450 nm on an enzyme immunoassay instrument. A cell viability >85% indicated the compounds were non-toxic, which were then examined in 5α-reductase inhibitory activity assay.

For the 5α-reductase inhibitory activity assay, HaCaT cells were seeded in 24-well plates (2 × 10^5^ cells/well) and cultured for 24 h. The wells were divided into control, positive (dutasteride) and experimental (tested samples at non-toxic concentration) groups, with 25 μM testosterones as a substrate and 250 μM NADPH in each well to culture for 24 h. Then the medium was removed and three volumes of ethyl acetate were added to form emulsion, which was centrifugated (10.000 rpm for 5 min at 20°C) and the ethyl acetate layer was dried and recovered in methanol.

### LC-MS determination of DHT

As described in the previously established method (Deng et al., 2020), LC-MS was performed using a LC-8050 triple-quadrupole MS (Shimadzu, Kyoto, Japan). The samples were analyzed on Waters-ACQUITY UPLC HSS T3 column (2.1 mm × 100 mm, 1.8 μm; Waters, United States) with a mobile phase consisting of 0.5 mM ammonium acetate in water and methanol (30:70, *v/v*). The flow rate was 0.4 ml/min, column temperature was 40°C and the injection volume was 5.0 μL. The MS was operated in the positive ion mode with an ESI source and the *m/z* 291.40 (DHT) was selected for detection in multiple reaction monitoring mode. The capillary and heater temperature were 300°C and 250°C respectively and the air flow rate was 10 L/min. The ratio of DHT content in the treated groups to that in the control group was calculated to evaluate the effects of DHT on the 5α-reductase activity.

## Results and discussion

### Optimization of *Lycium* carotenoid extraction

DESs have been widely used as green solvents to extract bioactive compounds from TCMs (Wang et al., 2016). To determine the most efficient *Lycium* carotenoids extraction method, the efficiency of existing representative methods (DESs extraction, traditional solvents extraction and supercritical fluid extraction) was evaluated by comparing the peak areas of zeaxanthin, ZD and total *Lycium* carotenoids. As shown in Supplementary Table S1, green extraction with DESs has more advantages because of the largest yields of zeaxanthin, ZD and total carotenoids, which were more than five times higher than those of the other two methods. What’s more, compared with the traditional solvent extraction method, the varieties and volumes of toxic organic solvents used in DESs-based extraction were substantially reduced, resulting in greater safety and environmental protection. Therefore, the DESs extraction method was used for sample preparation in the present study.

In order to obtain higher extraction efficiency, 13 DESs with varying solvent combinations and molar ratios were prepared and compared (Table 1). In addition, three methods for preparing DESs, including heating and stirring, freeze drying, and evaporating methods were further investigated (Fernández et al., 2018). Among the 13 DESs, DES-9 (choline chloride: malonic acid) presented the relatively high extraction yield rates both for zeaxanthin and ZD (Supplementary Figure S1), which could be supported by the fact that carotenoids are stable at acid environment, while saponification occurs when pH > 7.0 (Inbaraj et al., 2008). Furthermore, various molar ratios of these two reagents were examined (1:1 to 1:6; (Supplementary Figure S2), and it was observed that the extraction efficiency decreased with an increase of the malonic acid concentration and mixture viscosity. Obviously, the optimal molar ratio of choline chloride and malonic acid was 1:1, which was consistent with those of a previous study that declared the viscosity of the DESs was a more important factor for efficient extraction of bioactive compounds, with highly viscous DESs being unfavorable for extraction (Ali et al., 2019). Besides, the extraction conditions about ratios of sample to DESs (Supplementary Figure S3) and extraction times (Supplementary Figure S4) were also investigated and optimized, a sample to DES ratio of 1:2 (*w*/*w*) and ultrasound-assisted extraction duration of 4 hours were finally adopted.

### Optimization and comparison of chromatographic systems

The chromatographic conditions with the UPC^2^ system were optimized to obtain desirable resolutions for each carotenoid in the least time. As the column material was the most important factor in the supercritical CO_2_ UPC^2^ analysis, seven column (Milford, MA, United States) fillers were analyzed and compared including BEH (1.7 μm, 3.0 mm × 100 mm), Torus Diol (1.7 μm, 3.0 mm × 100 mm), CSH Fluoro-Phenyl (1.7 μm, 3.0 mm × 150 mm), BEH 2-EP (1.7 μm, 3.0 mm × 100 mm), Torus 1-AA (1.7 μm, 3.0 mm × 100 mm), Torus 2-PIC (1.7 μm, 3.0 mm × 100 mm) and HSS C_18_ SB (1.8 μm, 3.0 mm × 150 mm). As shown in Supplementary Figure S5, sample chromatograms with UPC^2^ HSS C_18_ SB column possessed more defined peak shapes and ideal separation degrees for each carotenoid. Above all, there was no obvious interference between the main component ZD and other trace components, which was consistent with the finding that the C_18_ stationary phases present acceptable selectivity for the separation of geometric isomers as a previous study reported (Rivera and Canela-Garayoa, 2012).

The CO_2_ co-solvents in the mobile phase are also important for sample analysis. Hence, 10 common and lower-toxicity organic solvents were examined, namely, acetonitrile - methanol (1:1, *v*/*v*; A), 0.1% ammonia in methanol (B), 0.1% formate in methanol (C), ethanol (D), acetonitrile (E), methanol (F), methanol–isopropanol (9:1, *v*/*v*; G), methanol–ethanol (7:3, *v*/*v*; H), methanol–ethanol (9:1, *v*/*v*; J), methanol–isopropanol (1:1, *v*/*v*; K) **(** Supplementary Figure S6). Methanol (F) was selected due to better separation degree as well as its less toxicity and simple preparation. Moreover, other optimized parameters, such as the back pressures (Supplementary Figure S7) and column temperatures (Supplementary Figure S8), did not contribute significantly to improving the separation degree but had an obvious change on the overall peak retention time. Therefore, desired separation could be achieved by combining with the above optimized parameters and adjusting the gradient of the mobile phase.

**FIGURE 1:**
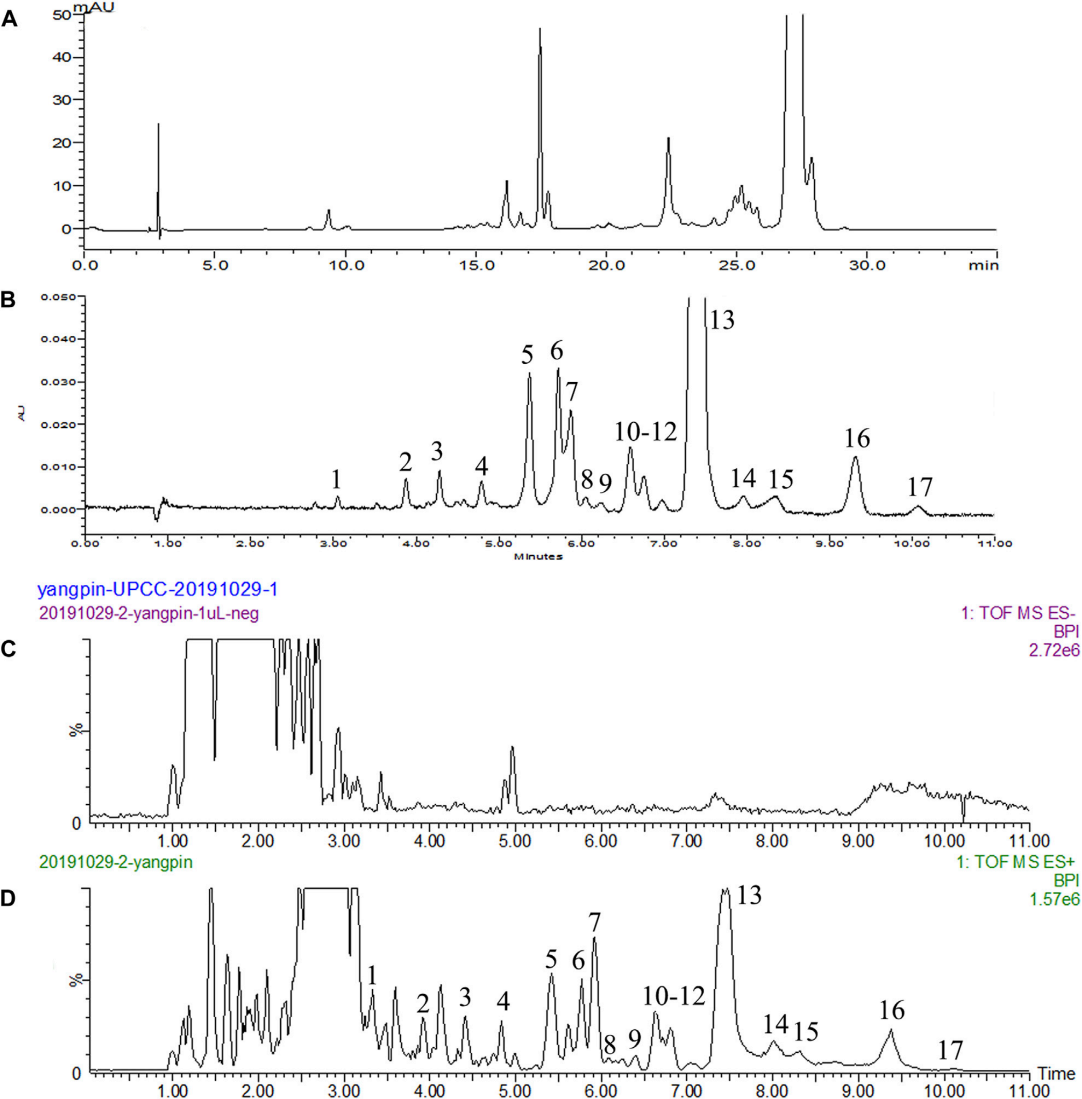
Typical chromatograms of *Lycium* carotenoids. **(A)** UPLC; **(B)** UPC^2^; **(C)** negative MS^E^; **(D)** positive MS^E^.

As shown in Figures 1A,B, the UPC^2^-PDA system performed better than the UPLC-PDA system for the characterization of *Lycium* carotenoids in terms of chemical resolution, chromatographic separation, adequate sensitivity to detect trace constituents and isomers as well as analysis time. An additional important advantage of the UPC^2^-PDA system was the replacement of the toxic solvents used in UPLC (acetonitrile and ethyl acetate) with environmentally friendly CO_2_ and methanol, resulting in more than ten-fold reduction in organic solvent usage. Therefore, the UPC^2^-PDA system is a more efficient and environmentally friendly method than UPLC-PDA system for chemical profiling of carotenoids from *L. barbarum* fruit.

### Identification and semi-quantification of *Lycium* carotenoids

The optimized UPC^2^-PDA analysis system was coupled with a Q-TOF-MS^E^ to determine the carotenoid composition of *L*. *barbarum* fruit. As opposed to negative ion mode (Figure 1C), the positive ion mode (Figure 1D) provided better mass spectra and fragment ion data for carotenoids. The MS^2^ fragment ion profiles and reported MS data for the identified carotenoids are provided in Table 2 and a total of seventeen carotenoids were detected, including one unknown component, two free carotenoids, and 14 carotenoid esters that were identified based on their elementary compositions. Additionally, in order to aid in chemical structure identification, the positions of the absorption maxima (λ_max_) of the seventeen components were determined, which showed most of them produce similar ultraviolet absorption spectra between 300 and 500 nm as carotenoids are composed of long polyisoprene chains with many conjugate double bonds.

**TABLE 2:** UPC^2^-PDA-Q-TOF-MS^E^ data of carotenoids and their esters in *L. barbarum* fruit.

Peak no	Rt (min)	Identity	λmax (nm)	m/z found	Content (μg/g)^c^
1	3.024	unknown	437	904.8334 [M + H]^+^	2.18 ± 0.29
2	3.848	All-*trans*-*β*-carotene^a^ ^,^ ^b^	435/464	537.4410 [M + H]^+^, 444.3745 [M-92]^+^	9.98 ± 0.37
3	4.253	All-*trans*-zeaxanthin^a^ ^,^ ^b^	439/466	569.4366 [M + H]^+^, 551.4283 [M + H-18]^+^, 476.3666 [M-92]^+^, 175.1452 [M-394]^+^	5.98 ± 0.42
4	4.737	Violaxanthin dipalmitate^b^	430/457	1077.8843 [M + H]^+^	3.46 ± 0.75
5	5.327	*β*-cryptoxanthin monopalmitate^b^	439/464	791.6636 [M + H]^+^, 535.4299 [M + H-PA]^+^	55.34 ± 0.62
6	5.677	Zeaxanthin monopalmitate^b^	440/467	807.6634 [M + H]^+^, 551.4254 [M + H-PA]^+^	44.99 ± 0.35
7	5.819	Antheraxanthin dipalmitate^b^	434/463	1061.8907 [M + H]^+^, 805.6508 [M + H-PA]^+^	25.62 ± 0.57
8	5.988	Antheraxanthin dipalmitate^b^	412/456	1061.8875 [M + H]^+^	1.61 ± 0.46
9	6.182	Antheraxanthin dipalmitate^b^	434/451	1061.8892 [M + H]^+^,	1.62 ± 0.22
10	6.529	Zeaxanthin myristate palmitate^b^	443/463	1017.8613 [M + H]^+^, 789.6548 [M + H-MA]^+^, 761.6204 [M + H-PA]^+^	19.06 ± 0.67
11	6.700	Zeaxanthin myristate palmitate^b^	437/472	1017.8612 [M + H]^+^, 789.6539 [M + H-MA]^+^, 761.6224 [M + H-PA]^+^	4.51 ± 0.63
12	6.905	Zeaxanthin myristate palmitate^b^	441/469	1017.8617 [M + H]^+^, 789.6541 [M + H-MA]^+^, 761.6216 [M + H-PA]^+^	1.82 ± 0.21
13	7.345	Zeaxanthin dipalmitate^a^ ^,^ ^b^	440/467	1045.8926 [M + H]^+^, 789.6537 [M + H-PA]^+^, 533.4143 [M + H-2PA]^+^	1315.46 ± 10.98
14	7.877	Zeaxanthin dipalmitate isomer Ⅰ^b^	438/463	1045.8907 [M + H]^+^, 789.6522 [M + H-PA]^+^, 533.4110 [M + H-2PA]^+^	4.57 ± 0.15
15	8.297	Zeaxanthin dipalmitate isomer Ⅱ^b^	432/466	1045.8909 [M + H]^+^, 789.6526 [M + H-PA]^+^, 533.4208 [M + H-2PA]^+^	5.53 ± 0.81
16	9.235	Zeaxanthin dipalmitate isomer Ⅲ^b^	434/464	1045.8917 [M + H]^+^, 789.6536 [M + H-PA]^+^, 533.4109 [M + H-2PA]^+^	25.96 ± 0.98
17	9.965	Zeaxanthin dipalmitate isomer Ⅳ^b^	443/461	1045.8934 [M + H]^+^, 789.6549 [M + H-PA]^+^, 533.4088 [M + H-2PA]^+^	4.23 ± 0.43

^a^ Identified by comparison with product ions of standard compound.

^b^ Based on reports by Inbaraj et al., 2008; Mertz et al., 2010; Crupi et al., 2010; Hempel et al., 2017.

^c^ The relative concentration of each carotenoid is reported as mean ± SD.

Rt, Retention time; MA, myristate acid; PA, palmitic acid.

**FIGURE 2:**
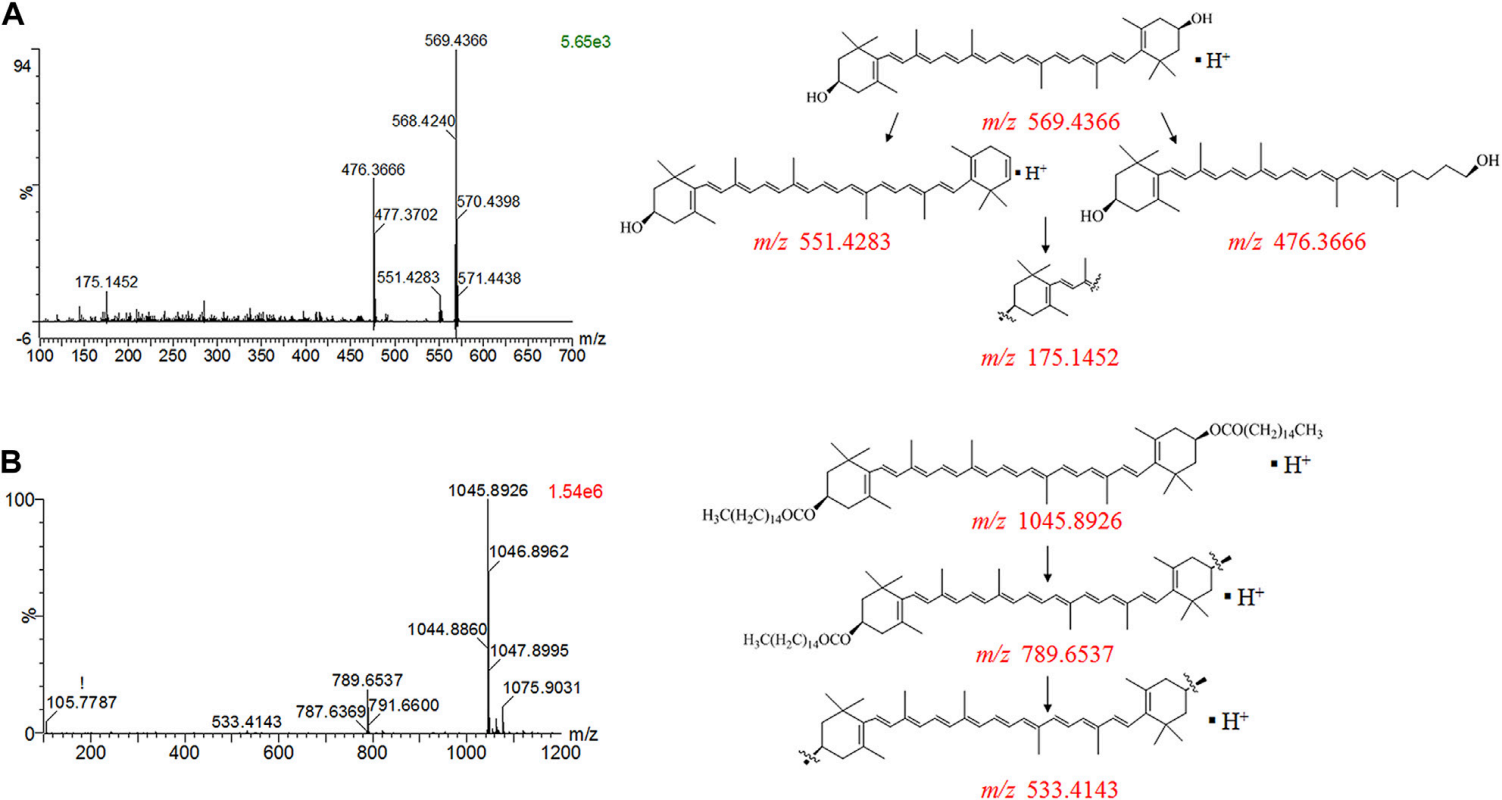
MS^E^ spectra of *Lycium* carotenoids. **(A)** Zeaxanthin; **(B)** zeaxanthin dipalmitate.

Peaks two and three were identified as all-*trans*-*β*-carotene and all-*trans*-zeaxanthin, respectively, which were typical free carotenoid components reported in *L. barbarum* fruit*.* In the MS spectra of all-*trans*-zeaxanthin (Figure 2A), the protonated molecular ion at *m/z* 569.4366 [M + H]^+^ represented a compound with the chemical formula of C_40_H_56_O_2_. Additionally, the main characteristic fragment ion [M−92]^+^ at *m/z* 476.3666 originated from in-chain fragmentation *via* the loss of toluene, a characteristic elimination of the polyene system, or the sequential loss of a 56 Da and two water molecules was also clearly observed for all-*trans*-*β*-carotene (peak 2) (Crupi et al., 2010). Peak 13 was the predominant component, covering more than 80% of the total peak area at 450 nm, and displayed a protonated ion at *m/z* 1045.8926 [M + H]^+^, which was tentatively identified as zeaxanthin ester by comparing with characteristic MS/MS fragments of standard substances and an ultraviolet (UV)—visible spectrum. Previous studies have reported carotenoid esters from *L. barbarum* fruit typically bind to one or two molecules of palmitic acid (PA), a fatty acid with a molecular weight of 256.4 g/mol, so peak 13 was identified as ZD according to the characteristic fragment ions at *m*/*z* 789.6537 [M + H-PA]^+^ and *m*/*z* 533.4143 [M + H-2PA]^+^ (Figure 2B) (Inbaraj et al., 2008; Long et al., 2019). Additionally, four peaks (14–17) were identified as ZD isomers, as their MS/MS fragmentation patterns and UV-visible spectra were similar to those of peak 13. Similarly, these fragmentation patterns were also observed in palmitate derivatives including violaxanthin (peak 4), *β*-cryptoxanthin (peak 5), zeaxanthin (peak 6), and antheraxanthin (peaks 7–9), while peaks 10, 11, and 12 were identified as zeaxanthin esters bound to one PA and one myristic acid (MA) ((Mertz et al., 2010; Hempel et al., 2017; Hempel et al., 2017). However, it is noteworthy that over half of the detected components could not be accurately identified, as they produced the same protonated molecules and similar fragment ion, which can be attributed to the complex chemical structures of carotenoids, with eight isoprenoid units and a conjugated double-bond system that results in numerous isomers. More importantly, carotenoids are highly susceptible to oxidation and *cis-trans* isomerization, especially when exposed to light, heat and acid, making it difficult to obtain high purity and stable isolated carotenoids to investigate their specific MS fragmentation patterns.

The contents of seventeen main *Lycium* carotenoids were semi-quantified (Table 2). As the results shown, the quantity of ZD (1,315.46 μg/g) was the highest, followed by *β*-cryptoxanthin monopalmitate (55.34 μg/g) and zeaxanthin monopalmitate (44.99 μg/g). However, the content of two free carotenoids, all-*trans*-*β*-carotene (9.98 μg/g) and all-*trans*-zeaxanthin (5.98 μg/g) was lower than that of their esters. Therefore, other applicable advanced separation and analysis techniques, such as preparative supercritical fluid chromatography should be evaluated in the future for their ability to obtain isolated *Lycium* carotenoids for characterization and quantification. Furthermore, our previous published article has been reported that the content of ZD was ranged from 0.09% to 0.32% on 26 batches of *L. barbarum* fruit from different producing areas in China, which was consisted with other reported results (Peng et al., 2005; Zhou et al., 2021; Xia et al., 2022). Although literature mentioned that the average content of ZD is 0.3%–0.4% through conventional extraction (Karioti et al., 2014), we thought this was mainly due to sample variation and the differences of growing environment, produced areas, harvest time, storage and transportation conditions of *L. barbarum* fruits were also the main factors affecting their content differences of ZD.

### Evaluation of 5α-reductase inhibitory activity

Increased 5α-reductase activity is a recognized diagnostic indicator of BPH and its inhibition can be evaluated by measuring DHT yield (Sun et al., 2011). Based on our previously developed method (Deng et al., 2020), the 5α-reductase inhibitory activity in HaCaT cells was determined by using LC-MS to monitor the conversion of testosterone into DHT, with dutasteride as the positive control. The ratio of DHT content in the treated samples to that in the control was used to evaluate the transformation potency, with the control transformation ratio set at 100%.

**FIGURE 3:**
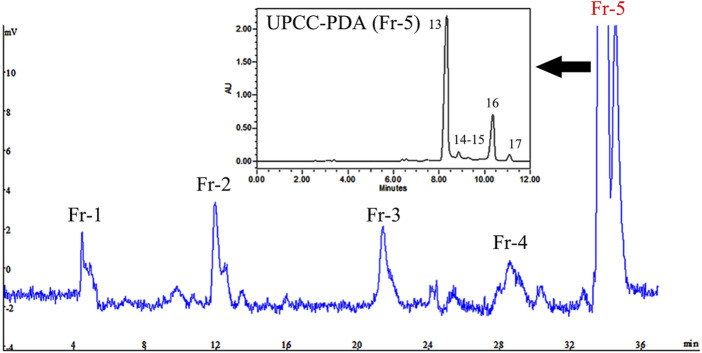
SPLC chromatogram of five different carotenoids fractions isolated from *L. barbarum* fruit and UPC^2^ chromatogram of Fr-5.

**FIGURE 4:**
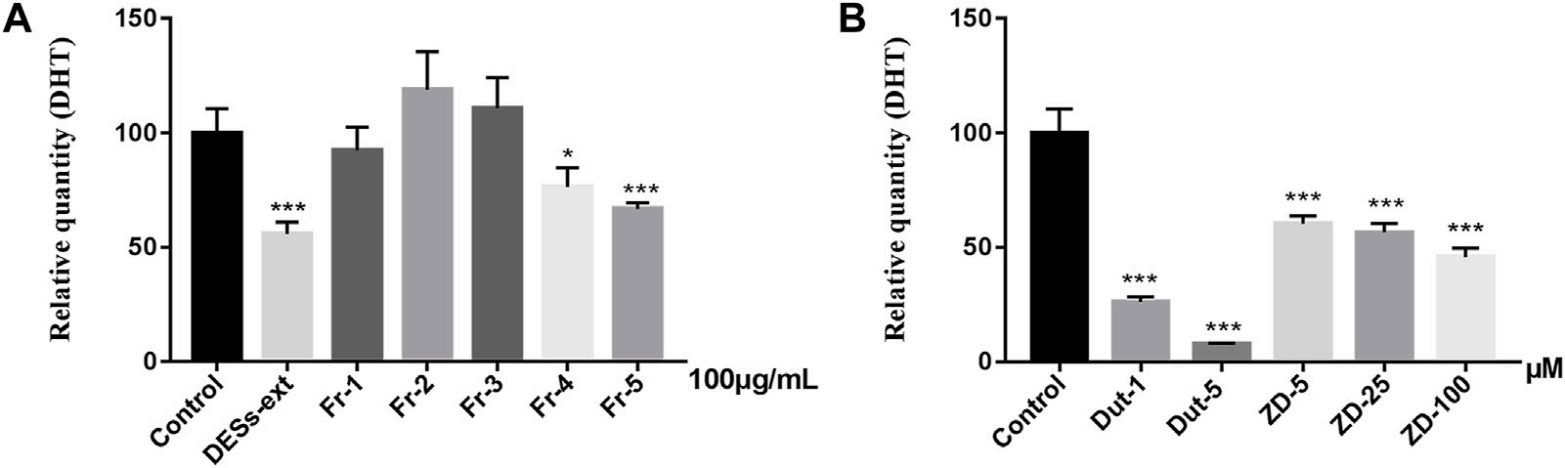
The relative quantity of DHT of five different carotenoids fractions **(A)** and zeaxanthin dipalmitate **(B)** (*n* = 6, Data are expressed as mean ± SD, *p < 0.05, **p < 0.01, ***p < 0.001 vs. control).

To obtain more potent fractions and components, DESs extracts were separated into five fractions (Fr-1 to Fr-5) according to marker distributions by SPLC and the fractions were also measured by UPC^2^-Q-TOF-MS^E^ (Figure 3). As shown in Supplementary Table S2, none of the extracts or fractions impaired cell viability at doses of 5, 12.5, 25, 50 and 100 μg/ml. Furthermore, the 5α-reductase inhibitory activity of *L. barbarum* fruit extracts and fractions were shown in Supplementary Table S3. At a non-toxic dose of 100 μg/ml, DESs extracts inhibited 5-reductase activity up to 44.24% while Fr-5 presented the most effective results among the fractions, with a transformation ratio of 66.84% at 100 μg/ml (Figure 4A). Subsequently, chemical profiling by UPC^2^-Q-TOF-MS^E^ revealed that Fr-5 was mainly composed of ZD (peak 13) and its isomers (peaks 14–17) (Figure 3).

ZD, the esterified and more stable form of zeaxanthin, represented more than 80% of the total *Lycium* carotenoids (Wang et al., 2015). To evaluate the potential activity of ZD, we purchased a commercially available >95% purity ZD. However, considering that ZD could not be fully dissolved in dimethyl sulfoxide for cell treatment as it was lipid-soluble pigment with extremely low polarity, several co-solvents or common surfactants, including Labrasol, Tween 80, Tween 20, Cremophor CO, Alkyl Polyglycoside (APG), and Poloxamer, were used to help dissolve or uniformly suspend ZD in the medium. The toxicity of these six substances at 0.1% concentration in DMEM was investigated using the cell viability assay (Supplementary Figure S9A) and Poloxamer was the least cytotoxic and could completely dissolve ZD. As shown in Supplementary Figure S9B, ZD did not show toxicity at 5, 25, and 100 μM concentrations and inhibited of the 5α-reductase activity dose dependently (Supplementary Table S3 and Figure 4B). Interestingly, these results suggested that directly chewing *L. barbarum* fruit could provide more carotenoids to patients with high DHT levels than brewing a tea or boiling a soup of the fruits. In the future, better purification of components such as peak 16 in Fr-5 should be achieved by pre-HPLC or pre-SFE for activity evaluation. In addition, differences and structure-activity relationships between free carotenoids and their esters require future investigation.

## Conclusion

In the present study, based on the concept of green chemistry, we firstly developed DESs extraction method couple with UPC^2^-Q-TOF-MS^E^ for the rapid analysis of carotenoid and marker compositions in *L. barbarum* fruit. The DESs containing choline chloride and malonic acid was optimal for the extraction of free carotenoids and their esters, with higher yields and better environmental protection than traditional organic solvent extraction. Compared with UPLC, carotenoids could be quickly separated and characterized within 11 min using UPC^2^-Q-TOF-MS^E^ and seventeen carotenoids were identified using MS/MS fragmentation patterns, among which ZD accounted for the highest. Furthermore, the *in vitro* 5α-reductase inhibitory activity of DESs extracts, fractions and components were compared, and the predominant component ZD exhibited particularly potent inhibitory activity. Our findings indicate that *L. barbarum* fruit are a rich source of this important carotenoids, and that their consumption may be useful for the prevention and treatment of BPH.

## Data Availability

The original contributions presented in the study are included in the article/Supplementary Material, further inquiries can be directed to the corresponding authors.

## Abbreviations

BPH, benign prostatic hyperplasia; DESs, deep eutectic solvents; DHT, dihydrotestosterone; DMEM, dulbecco’s modified Eagle medium; ESI, electrospray ionization; FBS, fetal bovine serum; HPLC, high-performance liquid chromatography; MS, mass spectrometry; NADPH, Nicotinamide adenine dinucleotide phosphate; PS, penicillin-streptomycin; Q-TOF-MS, quadrupole time-of-flight mass spectrometry; SFE, supercritical fluid extraction; SPLC, semi-preparative liquid chromatography; TCMs, traditional Chinese medicines; UPC^2^, ultra-performance convergence chromatography; UPC^2^-PDA-Q-TOF-MS^E^, ultra-performance convergence chromatography coupled with photo diode array detector and quadrupole time-of-flight mass spectrometry; UPLC, ultra-performance liquid chromatography; ZD, zeaxanthin dipalmitate.
