# Supplementary material for: Characterization of carotenoids in Lycium barbarum fruit by using UPC2-PDA-Q-TOF-MSE couple with deep eutectic solvents extraction and evaluation of their 5α-reductase inhibitory activity
Source: Front Chem. 2022 Nov 8;10:1052000. doi: 10.3389/fchem.2022.1052000 (PMC9679622; doi:10.3389/fchem.2022.1052000)
Supplement: Supplementary file 1 [file DataSheet1.docx]

**Supplementary Material**

Table S1. Comparison of different extraction methods of *Lycium* carotenoids

| No | Published methods | Peak areas | | |
| --- | --- | --- | --- | --- |
|  |  | Zeaxanthin | Zeaxanthin dipalmitate | Total |
| 1 | Traditional solvent extraction | 0 | 2123594 | 2294950 |
| 2 | Supercritical fluid extraction | 0 | 54880 | 141896 |
| 3 | DESs extraction | 53005 | 13822462 | 16669002 |

Table S2. Hacat cells viability of extract and fractions of *Lycium* carotenoids

| Name | Cell viability | | | | |
| --- | --- | --- | --- | --- | --- |
|  | 5μg/mL | 12.5μg/mL | 25μg/mL | 50μg/mL | 100μg/mL |
| DESs extract | 106.21±7.40 | 104.89±6.52 | 109.05±7.22 | 108.31±7.51 | 104.17±7.52 |
| Fr-1 | 120.94±10.63 | 98.90±8.60 | 102.31±9.28 | 104.97±9.34 | 106.53±10.61 |
| Fr-2 | 103.56±10.35 | 99.57±6.81 | 98.50±10.52 | 98.16±8.81 | 88.77±8.42 |
| Fr-3 | 97.34±10.16 | 98.39±10.09 | 94.62±10.34 | 91.86±9.61 | 87.19±9.10 |
| Fr-4 | 104.80±5.13 | 101.57±4.98 | 100.00±2.98 | 101.48±3.84 | 105.41±4.82 |
| Fr-5 | 111.02±9.20 | 109.98±4.33 | 103.34±10.37 | 96.82±9.81 | 103.63±10.68 |

Cell viability (% ): x= (OD _sample_－OD _blank_) / (OD _control_－OD _blank_); Data are expressed as mean ± SD

Table S3. 5α-reductase transformation and inhibition rate of extract, fractions and components of *Lycium* carotenoids

| Sample | Concentration | Transformation rate (%) | Inhibition rate (%) |
| --- | --- | --- | --- |
| Control | - | 100 | 0 |
| DESs extract | 100 μg/mL | 55.76 | 44.24 |
| Fr-1 | 100 μg/mL | 92.41 | 7.59 |
| Fr-2 | 100 μg/mL | 118.99 | -18.99 |
| Fr-3 | 100 μg/mL | 110.86 | -10.86 |
| Fr-4 | 100 μg/mL | 76.45 | 23.55 |
| Fr-5 | 100 μg/mL | 66.84 | 33.16 |
| ZD | 5 μM | 60.49 | 39.51 |
| ZD | 25 μM | 56.52 | 43.48 |
| ZD | 100 μM | 45.86 | 54.16 |
| Dutasteride | 1 μM | 26.21 | 73.79 |
| Dutasteride | 5 μM | 7.80 | 92.20 |

Data are expressed as ratio (%) at the sample non-toxic concentration; Transformation rate (%): x=C_a_/C_b_, Inhibition rate (%): y=1-x; C_a_: concentration (DHT) of samples group, C_b_: concentration (DHT) of control group.

Fig S1. The effect of different DESs on the *Lycium* carotenoids.

zeaxanthin **(A)**; zeaxanthin dipalmitate **(B)**

Fig S2. The effect of different proportion (Choline chloride: malonic acid) on *Lycium* carotenoids.

zeaxanthin **(A)**; zeaxanthin dipalmitate **(B)**


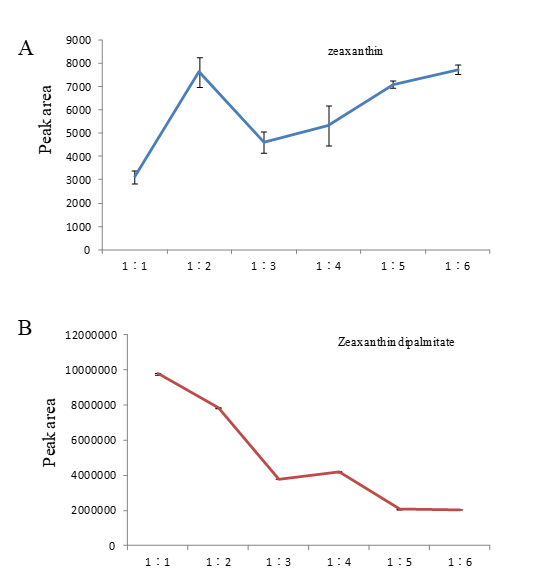


Fig S3. The effect of different sample to DES ratio (w/w) on *Lycium* carotenoids.

zeaxanthin **(A)**; zeaxanthin dipalmitate **(B)**


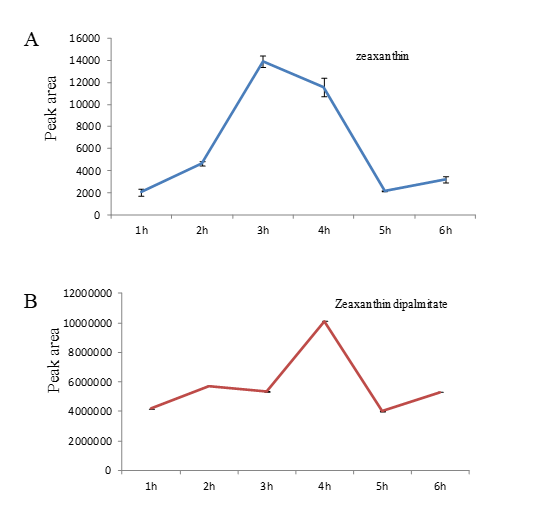


Fig S4. The effect of extracting time (h) on *Lycium* carotenoids.

Zeaxanthin **(A)**; zeaxanthin dipalmitate **(B)**


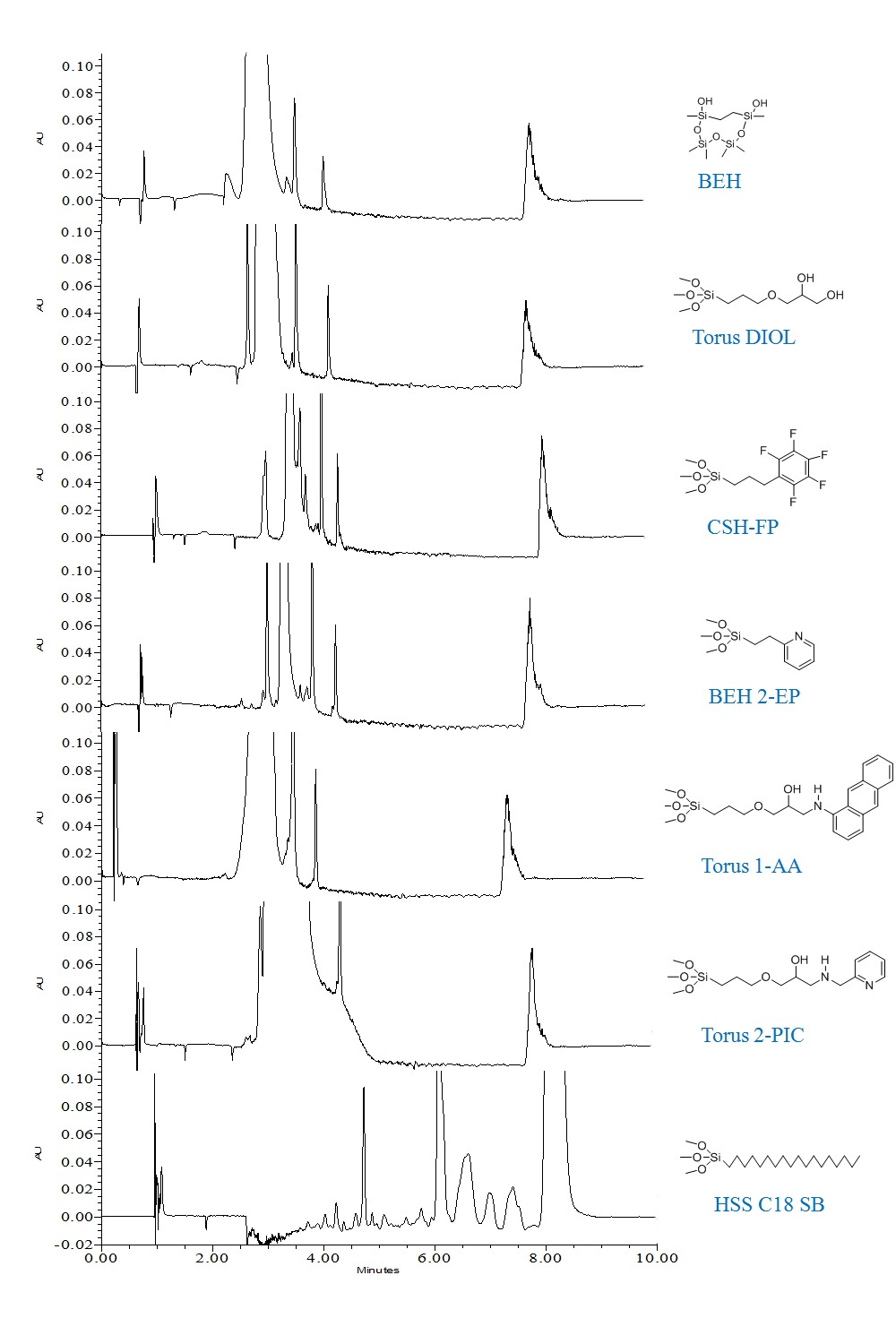


Fig S5. UPC^2^ chromatograms of *Lycium* carotenoids by using different columns.

Fig S6. UPC^2^ chromatograms for *Lycium* carotenoids by different co-solvent.

Acetonitrile - Methanol **(**1:1, **A)**; 0.1% Ammonia in Methanol **(B)**; 0.1% Formate in Methanol **(C)**; Ethanol **(D)**; Acetonitrile **(E)**; Methanol **(F)**; Methanol - Isopropanol **(**9:1, **G)**; Methanol - Ethanol **(**7:3, **H)**; Methanol - Ethanol **(**9:1, **J)**; Methanol - Isopropanol **(**1:1, **K)**

Fig S7. UPC^2^ chromatograms for *Lycium* carotenoids by different back pressure.

1500 psi **(A)**; 1600 psi **(B)**; 1800 psi **(C)**; 2000psi **(D)**

Fig S8. UPC^2^ chromatograms for *Lycium* carotenoids by different column temperature


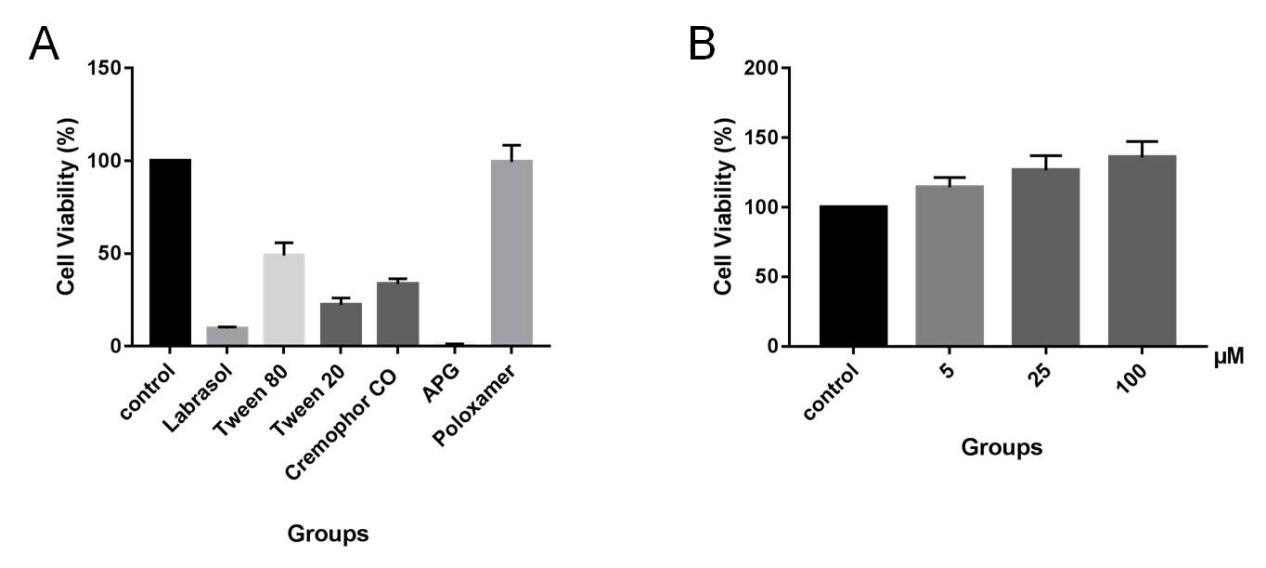


Fig S9. Hacat cells viability of different suspending agents **(A)** and zeaxanthin dipalmitate **(ZD, B)**
